# Medical and economic burden of delirium on hospitalization outcomes of acute respiratory failure: A retrospective national cohort

**DOI:** 10.1097/MD.0000000000032652

**Published:** 2023-01-13

**Authors:** Ahmed Taha, Huiping Xu, Roaa Ahmed, Ahmad Karim, John Meunier, Amal Paul, Ahmed Jawad, Manish L. Patel

**Affiliations:** a School of Medicine, Indiana University, Indianapolis, IN; b Department of Medicine, Deaconess Health System, Evansville, IN; c Department of Biostatistics and Health Data Science, School of Medicine, Indiana University, Indianapolis, IN; d School of Medicine, Ahfad University for Women, Omdurman, Sudan; e Department of Pulmonary and Critical Care, Deaconess Health System, Evansville, IN; f Division of Pulmonary & Critical Care, Texas Tech University Health Sciences Center, Amarillo, TX.

**Keywords:** cost of hospitalization, endotracheal intubation, healthcare expenditure, length of stay (LOS), mortality

## Abstract

Although delirium in patients with acute respiratory failure (ARF) may evolve in any hospital setting, previous studies on the impact of delirium on ARF were restricted to those in the intensive care unit (ICU). The data about the impact of delirium on ARF hospitalizations outside of the ICU is limited. Therefore, we conducted the first national study to examine the effect-magnitude of delirium on ARF in all hospital settings, that is, in the ICU as well as on the general medical floor. We searched the 2016 and 2017 National Inpatient Sample databases for ARF hospitalizations and created “Delirium” and “No delirium” groups. The outcomes of interest were mortality, endotracheal intubation, length of stay (LOS), and hospitalization costs. We also aimed to explore any potential demographic, racial, or healthcare disparities that may be associated with the diagnosis of delirium among ARF patients. Multivariable logistic regression was used to control for demographics and comorbidities. Delirium was present in 12.7% of the sample. Racial disparities among African Americans were also significant. Delirious patients had more comorbidities, higher mortality, and intubation rates (17.5% and 9.2% vs 10.6% and 6.1% in the “No delirium” group [*P* < .001], respectively). Delirious patients had a longer LOS and higher hospitalization costs (5.9 days and $15,395 USD vs 3.7 days and $9393 USD in “No delirium” [*P* < .001], respectively). Delirium was associated with worse mortality (adjusted odds ratio 1.49, confidence interval [CI] = 1.41, 1.57), higher intubation rates (adjusted odds ratio 1.46, CI = 1.36, 1.56), prolonged LOS (adjusted mean ratio 1.40, CI = 1.37, 1.42), and increased hospitalization costs (adjusted mean ratio 1.49, CI = 1.46, 1.52). A racial disparity in the diagnosis of delirium among African Americans hospitalized with ARF was noted in our sample. Patients in small, non-teaching hospitals were diagnosed with delirium less frequently compared to large, urban, teaching centers. Delirium predicts worse mortality and morbidity for ARF patients, regardless of bed placement and severity of the respiratory failure.

**Key points**

1. Delirium predicts worse mortality and morbidity in acute respiratory failure patients regardless of bed placement or severity of the respiratory failure.
2. Monitoring/prevention of delirium is advised for patients with acute respiratory failure in all hospital settings, not only in the intensive care unit.
3. Comparative and multi-center studies are needed to examine the racial disparity in the diagnosis of delirium among African Americans.

## 1. Introduction

As characterized by the American Psychiatric Association, delirium is an acute and transient disturbance in attention and cognition not explained by a preexisting neurocognitive disorder and is a direct consequence of a secondary medical condition such as hypoxia, metabolic derangement, substance intoxication/withdrawal, medication side-effect, etc.^[1]^ Delirium is linked to worse hospital outcomes and significant burden on the healthcare system – about $82.4 billion annually in the US – with adverse effects varying significantly according to the underlying diseases and studied population.^[2]^ However, delirium is a reversible condition; thus, hospital outcomes of primary medical illnesses are anticipated to improve if delirium is prevented and/or treated early.

Although delirium in patients with acute respiratory failure (ARF) may evolve in any hospital setting, previous studies on the impact of delirium on ARF were restricted to those in the intensive care unit (ICU). The data about the impact of delirium on ARF hospitalizations outside of the ICU is limited. Therefore, we conducted the first study, at a national scale, to examine the effect-magnitude of delirium on ARF in all hospital settings, that is, in the ICU as well as on the general medical floor. We also aimed to explore any potential demographic, racial, or healthcare disparities that may be associated with the diagnosis of delirium among ARF patients.^[3]^

## 2. Methods

### 2.1. Database and sample selection

The National Inpatient Sample (NIS) database of the Healthcare Cost and Utilization Project (HCUP) furnishes a de-identified sample of hospital discharges representative of >97% of the US population.^[4]^ We used the International Classification of Diseases, 10th Revision, Clinical Modification and Procedure Coding System to identify all hospitalizations with ARF in 2016 and 2017, (Table S1, Supplemental Digital Content, http://links.lww.com/MD/I323). We limited our sample to admissions where ARF was the primary diagnosis and delirium was reported as a complication or secondary diagnosis during the hospital course. To avoid reporting bias, encounters where ARF was reported as a secondary diagnosis were excluded.

### 2.2. Methodology

We used the complex survey design and NIS sampling weights – provided by HCUP – to obtain national estimates representative of the entire US population.^[5]^ The outcomes of interest were in-hospital mortality, rate of endotracheal intubation, length of stay (LOS), and total hospitalization cost.

Patient demographics, racial differences, hospital characteristics, and Elixhauser comorbidities were summarized using percentages for categorical variables and mean, with 95% confidence interval (CI), for continuous variables, (Table S2, Supplemental Digital Content, http://links.lww.com/MD/I324).^[6]^ Cost of hospitalization was estimated by multiplying hospital-specific cost-to-charge ratios with total hospital charges. Comparisons were performed using the Rao–Scott chi-square test for categorical variables and a survey-weighted linear regression for continuous variables. Outcomes were then summarized using percentages for binary variables and median, with interquartile range, for continuous variables due to their skewed distributions.

Multivariable logistic regression was then performed, (Tables S3–S6, Supplemental Digital Content, http://links.lww.com/MD/I325), and adjusted odds ratios (aORs) and corresponding 95% CI were calculated. A negative binomial regression was used for LOS while *γ* regression was used for hospitalization costs. A log link was used for both negative binomial and *γ* regression so that the exponentiated regression coefficients represent the ratio of the mean outcomes. The mean ratio (MR) and corresponding 95% CI were estimated for the effect of delirium. The linearity effect of continuous variables on individual outcomes was investigated using the dummy variable method, in which the continuous variable was categorized using tertiles, and the resulting dummy variables were included in the regression model. The *P* value was set at .05. All statistical analyses were performed using the survey procedures of SAS (version 9.4; SAS Institute, Cary, NC). This study is exempt from the Institutional Review Board because only de-identified data were used.

### 2.3. Exclusions

The following records were excluded:

1. Encounters of postoperative and post-traumatic respiratory failure, seizures and epilepsy, and drug-induced mental disorders were excluded to avoid reporting bias, that is, their ICD-10 codes can potentially interfere with those of ARF and/or delirium.
2. Records with pre-admission intubations were excluded because indication of intubation is unobtainable if it occurs prior to admission, and the rate of endotracheal intubation is an outcome of interest of the study.
3. Comorbidities with negligible prevalences (<1%) were excluded.
4. Records with missing data.
5. Only adults were included, so encounters with age <18 years were excluded.

## 3. Results

**Figure 1.**
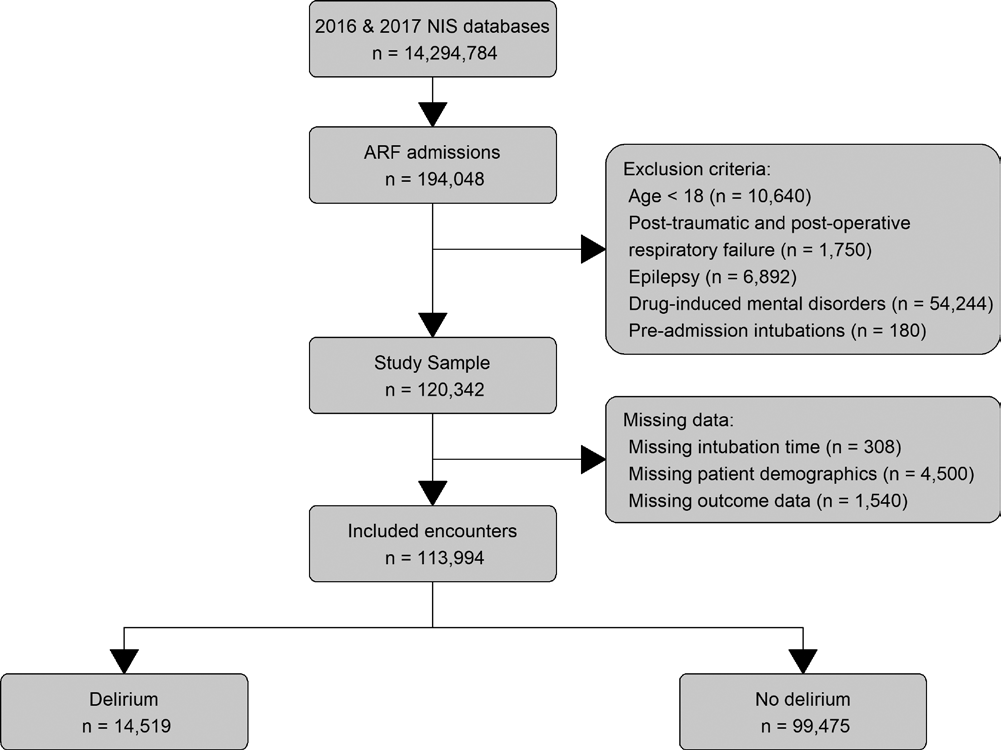
Study flow chart.

Of >14 million all-cause hospitalization records, we identified 113,994 records with a primary diagnosis of ARF that fulfilled the inclusion criteria, Figure 1. Delirium was present in 12.7% of the sample, mostly among Caucasians (73%), older patients (71.5 vs 68.9 years, *P* < .001), and Medicare beneficiaries (77.1% vs 71.8%, *P* < .001), Table 1. Delirium was diagnosed more frequently in large, urban, and teaching hospitals (63.5%) than in small non-teaching centers (54.3%).

**Table 1:** Patient demographics and hospital characteristics of acute respiratory failure hospitalizations in 2016 to 2017, by study group.

Variable	Total (n = 113,994)		*P* value
	Delirium (n = 14,519)	No delirium (n = 99,475)	
Age in yr, mean (95% CI)	71.5 (71.3, 71.6)	68.9 (68.8, 69.1)	<.001
<50	840 (5.8%)	8799 (8.8%)	
50–65	3252 (22.4%)	26622 (26.8%)	
>65	10427 (71.8%)	64054 (64.4%)	
Female sex	8239 (56.7%)	57257 (57.6%)	.061
Race			<.001
White	10601 (73.0%)	73724 (74.1%)	
Black	2281 (15.7%)	14480 (14.6%)	
Hispanic	926 (6.4%)	6864 (6.9%)	
Asian or Pacific Islander/Native American/Other	711 (4.9%)	4407 (4.4%)	
Primary payment source			<.001
Medicare	11197 (77.1%)	71383 (71.8%)	
Medicaid	1109 (7.6%)	9471 (9.5%)	
Private insurance	1634 (11.3%)	14062 (14.1%)	
Self-pay/No charge/Other	579 (4.0%)	4559 (4.6%)	
Hospital region			<.001
Northeast	2117 (14.6%)	16027 (16.1%)	
Midwest	3455 (23.8%)	23824 (23.9%)	
South	6483 (44.7%)	41922 (42.1%)	
West	2464 (17.0%)	17702 (17.8%)	
Hospital location and teaching status			<.001
Rural	1470 (10.1%)	13069 (13.1%)	
Urban nonteaching	3824 (26.3%)	28665 (28.8%)	
Urban teaching	9225 (63.5%)	57741 (58.0%)	
Hospital bed size			<.001
Small	2479 (17.1%)	20235 (20.3%)	
Medium	4163 (28.7%)	30871 (31.0%)	
Large	7877 (54.3%)	48369 (48.6%)	

CI = confidence interval.

Patient demographics – provided by the NIS database – are typically referenced to a control variable, that is, race is referenced to Caucasians and gender referenced to females^[7]^; therefore, we examined our sample for race, gender, and healthcare disparities. Gender and healthcare disparities in the sample were not statistically significant; however, a racial disparity for the diagnosis of delirium was significant among African Americans was significant (15.7% vs 14.6%, *P* < .001). Although the percentage difference was not large (15.7% vs 14.6% in the “No delirium” group), the *P* value was highly significant due to the extremely large sample size included.

**Figure 2.**
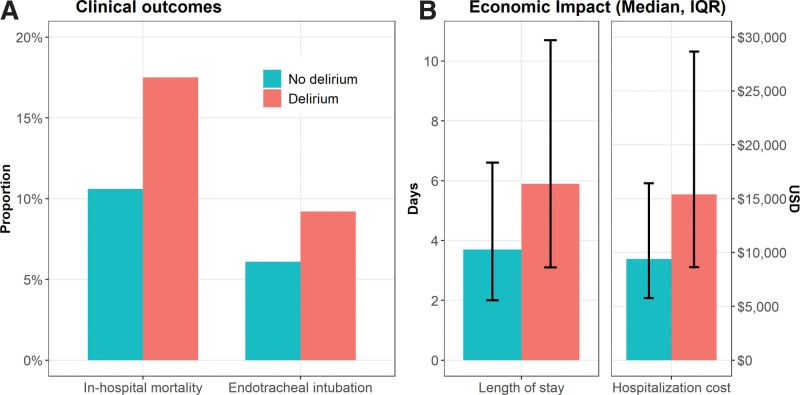
(A) Clinical and (B) economic outcomes of acute respiratory failure hospitalizations in 2016 to 2017, by study group.

Our survey also showed that ARF patients with delirium had more comorbidities and were sicker compared to those without delirium, Table 2. In line with current literature, the hospital mortality and rate of intubations were significantly worse among patients with ARF and delirium (17.5% and 9.2% vs 10.6% and 6.1% ARF in the “No Delirium” group [*P* < .001], respectively), Figure 2A. The median LOS and cost of hospitalization were also notably higher among patients with delirium (5.9 days and $15,395 USD vs 3.7 days and $9393 USD in the “No delirium” group, respectively [*P* < .001]), Figure 2B.

**Table 2:** Incidence of relevant comorbidities for acute respiratory failure hospitalizations in 2016 to 2017, by study group.

Variable	Total (n = 113,994)		*P* value
	Delirium (n = 14,519)	No delirium (n = 99,475)	
Anemia	4794 (33.0%)	25392 (25.5%)	<.001
Autoimmune conditions	565 (3.9%)	4562 (4.6%)	<.001
Cancer	1065 (7.3%)	7828 (7.9%)	<.001
Cerebrovascular disease	1276 (8.8%)	4062 (4.1%)	<.001
Heart failure	7618 (52.5%)	43957 (44.2%)	<.001
Coagulopathy	1851 (12.7%)	7312 (7.4%)	<.001
Dementia	2917 (20.1%)	7157 (7.2%)	<.001
Depression	2267 (15.6%)	14776 (14.9%)	.017
Diabetes	6360 (43.8%)	38245 (38.4%)	<.001
Hypertension	11088 (76.4%)	73010 (73.4%)	<.001
Liver disease	682 (4.7%)	3732 (3.8%)	<.001
Chronic pulmonary disease	8848 (60.9%)	70779 (71.2%)	<.001
Obesity	4510 (31.1%)	27160 (27.3%)	<.001
Paralysis	1048 (7.2%)	3627 (3.6%)	<.001
Peripheral vascular disease	1203 (8.3%)	7876 (7.9%)	.13
Psychoses	881 (6.1%)	3803 (3.8%)	<.001
Pulmonary circulation disease	2335 (16.1%)	15720 (15.8%)	.39
Renal disease	4760 (32.8%)	25071 (25.2%)	<.001
Thyroid disorder	2813 (19.4%)	18081 (18.2%)	<.001
Valvular disease	1528 (10.5%)	10088 (10.1%)	.16
Weight loss	2239 (15.4%)	9123 (9.2%)	<.001

**Figure 3.**
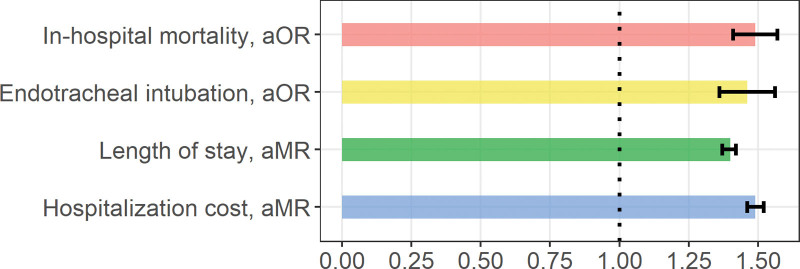
Impact of delirium on acute respiratory failure hospitalization outcomes in 2016 to 2017.

The multivariate analysis indicated that delirium is associated with greater odds of mortality (aOR 1.49, CI = 1.41, 1.57), endotracheal intubation (aOR 1.46, CI = 1.36, 1.56), prolonged LOS (adjusted MR 1.40, CI = 1.37, 1.42), and increased cost of hospitalization (adjusted MR 1.49, CI = 1.46, 1.52), Figure 3.

## 4. Discussion

The prevalence of delirium among ARF hospitalizations, regardless of bed placement, was 12.7%. Most ARF patients who developed delirium were Caucasian (73%) and aged >65 years (71.8%). Given that delirium is often under-recognized – one study showed delirium can be missed 64.5% of the time^[8]^ – the actual prevalence of delirium among ARF patients (in all hospital settings) is anticipated to be higher than our reported result.

Our survey indicated that patients in small, non-teaching hospitals were diagnosed with delirium less frequently compared to large, urban, teaching centers. Hospital’s teaching status was also associated with higher hospitalization costs, likely because of the higher level of disease acuity and the increased number of tests/procedures commonly performed in academic centers to identify an underlying etiology for delirium or to rule out an alternative diagnosis, such as lumbar puncture, electroencephalogram, etc.^[9]^ Current data, however, did not show any significant mortality or morbidity differences related to the significant difference in healthcare expenditures in large teaching centers.^[10]^

The available literature about racial disparity in the diagnosis of delirium is limited. Our survey is the first to demonstrate significant racial disparity among African Americans for the diagnosis of delirium at a national level. Only 2 single-center studies pertinent to the racial disparity of delirium among African Americans were previously published. Campbell et al demonstrated a racial disparity in the documentation of cognitive impairment among African Americans,^[11]^ and, in another single-center study, Khan et al indicated no association between the development of ICU-delirium and African American race.^[12]^

It is imperative to note that these 2 single-center studies were conducted in an urban metro area (Indianapolis, IN) where the pooled results are directly affected by patients’ demographics, social practices, and prevalence of chronic diseases in the local community where the study was conducted. Our cohort, on the other hand, involved a larger sample size involving the entire US, hence, not affected by local demographic or geographic differences (inherent to single-center studies); therefore, the racial disparity reported in our survey is more representative of the US population. Above all, this reported racial disparity in the diagnosis of delirium among African Americans should be interpreted with caution. It may represent a true and significant nation-wide epidemiological finding, a racial bias among healthcare providers, or a simple social discrepancy between races. Therefore, comparative prospective studies that control all socio-economic differences and analyze biological race as the sole variable are needed to explore this relationship.

Our study is also the first to quantify the burden of delirium on ARF for the entirety of hospitalization, that is, in the ICU and general medical floor. Our study showed that delirium is associated with a 90% increase in overall hospital mortality, 70% increase in intubation rate, 59.5% increase in LOS, and 64% increase in hospitalization costs, Figure 2. In line with current literature that associates delirium with a multitude of adverse clinical and hospital outcomes, our survey showed that ARF patients with delirium had significantly worse mortality, morbidity, and a higher economic burden than those without delirium.

The multivariate analysis revealed that delirium was independently associated with 49% greater odds for increased mortality, 46% increased risk for endotracheal intubation, 40% increased risk for longer LOS, and 49% increased risk for a higher hospitalization cost, Figure 3. These findings emphasize the importance of monitoring, prevention, and early management of delirium in all patients admitted with ARF, regardless of the hospital setting, bed placement, or the severity of the respiratory failure. Beyond its clinical benefits, measures to prevent delirium (i.e., non-pharmacological and multidisciplinary protocols) can also be perceived as strategies to reduce the economic burden of ARF hospitalization in terms of LOS, hospitalization cost, and post-discharge nursing home placement.^[13,14]^

## 5. Strengths and limitations

The NIS yielded enormous statistical power in this study by capturing significantly larger ARF observations compared to any previously published study. Our data and conclusions are also generalizable to the entire US population. This study had several limitations. Errors in coding, variations in billing, and discrepancies in physician documentation can influence the accurate assignment of ICD-10 codes and eventually lead to inaccuracies in estimating prevalences and outcomes. To improve the accuracy, we used ICD-10 codes that have been validated by and remain consistent with previously published literature.^[15]^

The NIS also lacks some important physiological data related to ARF outcomes, such as disease severity, Sequential Organ Failure Assessment score, and long-term outcomes. In addition, several concurrent diagnoses may have confounded the outcomes. For example, sepsis and pneumonia can cause both ARF and delirium and are also associated with higher mortality and longer LOS in patients with ARF.^[16]^ We mitigated this confounding bias by using a multivariable analysis with adjustment for all comorbidities provided by HCUP, as well as patient demographics and hospital characteristics, (Tables S3–S6, Supplemental Digital Content, http://links.lww.com/MD/I325).

## 6. Conclusions

Our national estimates provide strong population-based results – not previously available – on the clinical and economic outcomes of delirium among ARF hospitalizations that can be utilized for comparative and multi-center studies. Our study concluded that delirium remains a significant and independent predictor of worse hospital outcomes and increased healthcare burden among ARF hospitalizations; prevention and early management of delirium in ARF are advised in all hospital settings, regardless of bed placement or the severity of the underlying respiratory failure; and a significant racial disparity in the diagnosis of delirium was noted among African Americans hospitalized with ARF, its clinical relevance is not clear though.

## Author contributions

AT designed and conceptualized the study; performed the acquisition, analysis, and interpretation of the data; drafted and critically revised the manuscript. HX performed data analysis and drafted the methodology. RA, JM, AK, and AP reviewed the literature and wrote the first draft of the manuscript. AJ and MLP interpreted the data and critically revised the manuscript.

**Conceptualization:** Ahmed Taha, Ahmed Jawad, Manish L. Patel.

**Data curation:** Ahmed Taha, Huiping Xu.

**Formal analysis:** Huiping Xu.

**Methodology:** Huiping Xu.

**Software:** Huiping Xu.

**Supervision:** Ahmed Taha, Ahmed Jawad, Manish L. Patel.

**Writing – original draft:** Ahmed Taha, Roaa Ahmed, Ahmad Karim, John Meunier, Amal Paul, Ahmed Jawad, Manish L. Patel.

**Writing – review & editing:** Ahmed Taha, Ahmed Jawad, Manish L. Patel.

## Supplementary Material


