# Supplementary material for: Medical and economic burden of delirium on hospitalization outcomes of acute respiratory failure: A retrospective national cohort
Source: Medicine (Baltimore). 2023 Jan 13;102(2):e32652. doi: 10.1097/MD.0000000000032652 (PMC9839276; doi:10.1097/MD.0000000000032652)
Supplement: Supplementary file 1 [file medi-102-e32652-s001.pdf]

Supplemental Digital Content (Table S1): International Classification of Diseases, 10th Revision, Clinical Modification and Procedure Coding System (ICD-10 CM/PCS) Codes That Were Used.

**Diagnosis/ Procedure**	**ICD-10 CM/PCS code**
**1.** Delirium	R41.x*, R41.81, F01.51, F02.81, F03.91, F05.x*, F06.x*, F06.2, F06.8, G93.4, G93.49, G93.41, G92.9, G92.8
2. Acute respiratory failure	J96.0, J96.01, J96.02, J96.20, J96.22, J96.21, J80
3. Intubation	5A1935Z, 5A0945Z
**4.** Prolonged intubation (>96 hours)	5A0955Z

DIAGNOSES OF EXCLUSION

Pre-admission intubations	Day of intubation <0 relative to admission.
Post-traumatic and post-operative respiratory failure	J95
Epilepsy	G40.909
Drug-induced mental disorders	F10.x-F19.x*

*Abbreviations: .x: including subcodes, .00-.99
