# Supplementary material for: Medical and economic burden of delirium on hospitalization outcomes of acute respiratory failure: A retrospective national cohort
Source: Medicine (Baltimore). 2023 Jan 13;102(2):e32652. doi: 10.1097/MD.0000000000032652 (PMC9839276; doi:10.1097/MD.0000000000032652)
Supplement: Supplementary file 2 [file medi-102-e32652-s002.pdf]

Supplemental Digital Content (Table S2): Incidence of Comorbidities for Acute Respiratory Failure Hospitalizations in 2016-2017, according to HCUP Elixhauser Comorbidity Index (v2022.1), by Study Group.

Comorbidity Measure	Data Element Name	Delirium	No delirium
1. Acquired immune deficiency syndrome	CMR_AIDS	43 (0.3%)	447 (0.4%)
2. Alcohol abuse	CMR_ALCOHOL	12 (0.1%)	54 (0.1%)
3. Anemia	CMR_ANEMDEF, CMR_BLDLOSS	5068 (33.1%)	26835 (25.6%)
4. Autoimmune conditions	CMR_AUTOIMMUNE	595 (3.9%)	4821 (4.6%)
5. Lymphoma/Leukemia	CMR_CANCER_LYMPH, CMR_CANCER_LEUK	268 (1.8%)	1745 (1.7%)
6. Cancer	CMR_CANCER_METS, CMR_CANCER_NSITU, CMR_CANCER_SOLID	1135 (7.4%)	8230 (7.9%)
7. Cerebrovascular disease	CMR_CBVD	1340 (8.8%)	4336 (4.1%)
8. Heart failure	CMR_HF	8016 (52.4%)	46280 (44.2%)
9. Coagulopathy	CMR_COAG	1970 (12.9%)	7745 (7.4%)
10. Dementia	CMR_DEMENTIA	3060 (20.0%)	7515 (7.2%)
11. Depression	CMR_DEPRESS	2394 (15.7%)	15576 (14.9%)
12. Diabetes	CMR_DIAB_UNCX, CMR_DIAB_CX	6674 (43.7%)	40269 (38.4%)
13. Hypertension	CMR_HTN_CX, CMR_HTN_UNCX	11648 (76.2%)	76774 (73.3%)
14. Liver disease	CMR_LIVER_MLD, CMR_LIVER_SEV	718 (4.7%)	3950 (3.8%)
15. Chronic pulmonary disease	CMR_LUNG_CHRONIC	9276 (60.7%)	74137 (70.8%)
16. Neurological disorders	CMR_NEURO_MOVT, CMR_NEURO_OTH	13951 (91.2%)	5667 (5.4%)
17. Seizures and epilepsy	CMR_NEURO_SEIZ	843 (5.5%)	2001 (1.9%)
18. Obesity	CMR_OBESE	4747 (31.0%)	28637 (27.3%)
19. Paralysis	CMR_PARALYSIS	1103 (7.2%)	3855 (3.7%)
20. Peripheral vascular disease	CMR_PERIVASC	1309 (8.6%)	8610 (8.2%)
21. Psychoses	CMR_PSYCHOSES	931 (6.1%)	4035 (3.9%)
22. Pulmonary circulation disease	CMR_PULMCIRC	2457 (16.1%)	16660 (15.9%)
23. Renal disease	CMR_RENLFL_MOD, CMR_RENLFL_SEV	5025 (32.9%)	26553 (25.4%)
24. Thyroid disorder	CMR_THYROID_HYPO, CMR_THYROID_OTH	2948 (19.3%)	19072 (18.2%)
25. Peptic ulcer disease x bleeding	CMR_ULCER_PEPTIC	161 (1.1%)	835 (0.8%)
26. Valvular disease	CMR_VALVE	1605 (10.5%)	10671 (10.2%)
27. Weight loss	CMR_WGHTLOSS	2415 (15.8%)	9822 (9.4%)
