# Supplementary material for: Medical and economic burden of delirium on hospitalization outcomes of acute respiratory failure: A retrospective national cohort
Source: Medicine (Baltimore). 2023 Jan 13;102(2):e32652. doi: 10.1097/MD.0000000000032652 (PMC9839276; doi:10.1097/MD.0000000000032652)
Supplement: Supplementary file 3 [file medi-102-e32652-s003.pdf]

Supplemental Digital Content (Table S3): Predictors of All-Cause In-Hospital Mortality in Acute Respiratory Failure Hospitalizations with Delirium.

Variable	Univariable analysis	*p*-value	Multivariable analysis	*p*-value
	OR (95% CI)		aOR (95% CI)	
vs. No delirium group	1.80 (1.71, 1.89)	<0.001	1.49 (1.41, 1.57)	<0.001
Patient demographics				
Age	1.021 (1.020, 1.023)	<0.001	1.025 (1.022, 1.027)	<0.001
Female (vs male)	0.77 (0.75, 0.80)	<0.001	0.89 (0.86, 0.93)	<0.001
Race				
White	Reference		Reference	
Black	0.90 (0.85, 0.95)	<0.001	0.94 (0.88, 1)	0.056
Hispanic	1.12 (1.04, 1.21)	0.002	1.01 (0.94, 1.1)	0.73
Asian or Pacific Islander/Native American/Other	1.30 (1.20, 1.42)	<0.001	1.03 (0.95, 1.13)	0.45
Primary payment source				
Medicare	Reference		Reference	
Medicaid	0.68 (0.63, 0.73)	<0.001	1.11 (1.02, 1.21)	0.018
Private insurance	1.28 (1.20, 1.36)	<0.001	1.55 (1.44, 1.66)	<0.001
Self-pay/No charge/Other	1.89 (1.71, 2.09)	<0.001	2.36 (2.11, 2.63)	<0.001
Hospital characteristics				
Hospital region				
Northeast	Reference		Reference	
Midwest	0.82 (0.76, 0.88)	<0.001	0.87 (0.8, 0.94)	<0.001
South	0.89 (0.83, 0.95)	<0.001	0.89 (0.83, 0.96)	0.001
West	0.95 (0.88, 1.02)	0.15	0.87 (0.81, 0.94)	<0.001
Hospital location and teaching status				
Rural	Reference			
Urban nonteaching	1.39 (1.28, 1.50)	<0.001	1.33 (1.22, 1.44)	<0.001
Urban teaching	1.64 (1.52, 1.77)	<0.001	1.51 (1.4, 1.64)	<0.001
Hospital bed size				
Small	Reference		Reference	
Medium	1.12 (1.05, 1.20)	<0.001	1.12 (1.05, 1.2)	<0.001
Large	1.23 (1.15, 1.30)	<0.001	1.25 (1.18, 1.34)	<0.001
Comorbidities				
Anemia	1.06 (1.01, 1.10)	0.011	0.87 (0.83, 0.91)	<0.001
Autoimmune conditions	0.89 (0.81, 0.98)	0.015	0.94 (0.85, 1.03)	0.17
Cancer	2.64 (2.51, 2.79)	<0.001	2.18 (2.06, 2.31)	<0.001
Cerebrovascular disease	2.00 (1.86, 2.15)	<0.001	1.71 (1.55, 1.88)	<0.001
Heart failure	1.24 (1.20, 1.29)	<0.001	1.32 (1.27, 1.38)	<0.001
Coagulopathy	2.39 (2.27, 2.53)	<0.001	1.84 (1.73, 1.95)	<0.001
Dementia	1.56 (1.48, 1.65)	<0.001	1.08 (1.01, 1.15)	0.027
Depression	0.57 (0.54, 0.61)	<0.001	0.69 (0.64, 0.73)	<0.001
Diabetes	0.85 (0.82, 0.89)	<0.001	0.96 (0.92, 1)	0.045
Hypertension	0.75 (0.72, 0.78)	<0.001	0.71 (0.68, 0.75)	<0.001
Liver disease	1.50 (1.38, 1.63)	<0.001	1.3 (1.19, 1.42)	<0.001

Chronic pulmonary disease	0.35 (0.33, 0.36)	<0.001	0.4 (0.39, 0.42)	<0.001
Obesity	0.51 (0.49, 0.54)	<0.001	0.68 (0.64, 0.72)	<0.001
Paralysis	1.37 (1.25, 1.49)	<0.001	0.83 (0.73, 0.93)	0.001
Peripheral vascular disease	1.07 (1.00, 1.14)	0.049	0.99 (0.93, 1.07)	0.89
Psychoses	0.56 (0.50, 0.63)	<0.001	0.75 (0.66, 0.84)	<0.001
Pulmonary circulation disease	0.92 (0.88, 0.97)	0.004	0.99 (0.93, 1.05)	0.69
Renal disease	1.25 (1.20, 1.30)	<0.001	1.15 (1.09, 1.2)	<0.001
Thyroid disorder	0.83 (0.79, 0.87)	<0.001	0.83 (0.79, 0.88)	<0.001
Valvular disease	1.16 (1.09, 1.23)	<0.001	0.9 (0.84, 0.96)	<0.001
Weight loss	1.86 (1.77, 1.96)	<0.001	1.42 (1.34, 1.5)	<0.001

Supplemental Digital Content (Table S4): Predictors of Endotracheal Intubations in Acute Respiratory Failure Hospitalizations with Delirium.

Variable	Univariable analysis	*p*-value	Multivariable analysis	*p*-value
	OR (95% CI)		aOR (95% CI)	
vs. No delirium	1.56 (1.46, 1.66)	<0.001	1.46 (1.36, 1.56)	<0.001
Patient demographics				
Age	0.990 (0.989, 0.992)	<0.001	0.989 (0.987, 0.991)	<0.001
Female vs male	0.84 (0.80, 0.88)	<0.001	0.95 (0.9, 1)	0.034
Race				
White	Reference		Reference	
Black	1.26 (1.18, 1.35)	<0.001	1.09 (1.01, 1.17)	0.021
Hispanic	1.39 (1.27, 1.51)	<0.001	1.14 (1.04, 1.25)	0.007
Asian or Pacific Islander/Native American/Other	1.42 (1.27, 1.58)	<0.001	1.17 (1.05, 1.3)	0.005
Primary payment source				
Medicare	Reference		Reference	
Medicaid	1.16 (1.07, 1.25)	<0.001	0.9 (0.82, 0.99)	0.023
Private insurance	1.15 (1.08, 1.24)	<0.001	0.91 (0.85, 0.98)	0.015
Self-pay/No charge/Other	1.45 (1.31, 1.61)	<0.001	1.11 (0.99, 1.24)	0.063
Hospital characteristics				
Hospital region				
Northeast	Reference		Reference	
Midwest	0.89 (0.81, 0.97)	0.011	0.94 (0.85, 1.02)	0.14
South	0.99 (0.92, 1.08)	0.89	0.95 (0.87, 1.03)	0.19
West	0.95 (0.87, 1.04)	0.26	0.88 (0.81, 0.97)	0.007
Hospital location and teaching status				
Rural	Reference		Reference	
Urban nonteaching	1.31 (1.18, 1.45)	<0.001	1.29 (1.16, 1.43)	<0.001
Urban teaching	1.59 (1.45, 1.76)	<0.001	1.48 (1.34, 1.63)	<0.001
Hospital bed size				
Small	Reference		Reference	
Medium	1.19 (1.10, 1.29)	<0.001	1.17 (1.08, 1.27)	<0.001
Large	1.25 (1.16, 1.35)	<0.001	1.24 (1.15, 1.33)	<0.001
Comorbidities				
Anemia	1.01 (0.95, 1.06)	0.79	0.9 (0.85, 0.96)	<0.001
Autoimmune conditions	0.82 (0.73, 0.93)	0.002	0.83 (0.73, 0.94)	0.003
Cancer	1.16 (1.07, 1.26)	<0.001	1.06 (0.97, 1.15)	0.22
Cerebrovascular disease	1.47 (1.33, 1.62)	<0.001	1.32 (1.17, 1.49)	<0.001
Heart failure	1.14 (1.09, 1.20)	<0.001	1.29 (1.22, 1.36)	<0.001
Coagulopathy	1.34 (1.24, 1.45)	<0.001	1.12 (1.03, 1.21)	0.007
Dementia	0.99 (0.91, 1.08)	0.84	0.94 (0.86, 1.02)	0.16
Depression	0.75 (0.70, 0.81)	<0.001	0.84 (0.78, 0.9)	<0.001
Diabetes	1.06 (1.01, 1.12)	0.012	1.08 (1.02, 1.14)	0.007
Hypertension	0.84 (0.80, 0.89)	<0.001	0.88 (0.83, 0.93)	<0.001

Liver disease	1.32 (1.19, 1.48)	<0.001	1.12 (1, 1.25)	0.052
Chronic pulmonary disease	0.43 (0.41, 0.45)	<0.001	0.47 (0.45, 0.5)	<0.001
Obesity	0.76 (0.72, 0.80)	<0.001	0.72 (0.67, 0.76)	<0.001
Paralysis	1.24 (1.11, 1.39)	<0.001	0.79 (0.69, 0.91)	<0.001
Peripheral vascular disease	1.01 (0.92, 1.10)	0.87	1.06 (0.97, 1.17)	0.19
Psychoses	1.05 (0.93, 1.18)	0.42	1.04 (0.92, 1.17)	0.54
Pulmonary circulation disease	0.69 (0.64, 0.75)	<0.001	0.69 (0.64, 0.75)	<0.001
Renal disease	1.13 (1.07, 1.19)	<0.001	1 (0.94, 1.06)	0.96
Thyroid disorder	0.77 (0.72, 0.82)	<0.001	0.83 (0.78, 0.89)	<0.001
Valvular disease	1.13 (1.05, 1.22)	0.001	1.14 (1.06, 1.24)	<0.001
Weight loss	0.89 (0.82, 0.97)	0.006	0.8 (0.74, 0.87)	<0.001

Supplemental Digital Content (Table S5): Predictors of Increased Length of Stay for Acute Respiratory Failure Hospitalizations with Delirium.

Variable	Univariable analysis	*p*-value	Multivariable analysis	*p*-value
	IRR (95% CI)		aIRR (95% CI)	
vs. No delirium	1.56 (1.53, 1.59)	<0.001	1.4 (1.37, 1.42)	<0.001
Patient demographics				
Age	0.996 (0.995, 0.996)	<0.001	0.996 (0.995, 0.997)	<0.001
Female vs male	0.96 (0.95, 0.98)	<0.001	0.98 (0.96, 0.99)	<0.001
Race				
White	Reference		Reference	
Black	1.19 (1.16, 1.22)	<0.001	1.04 (1.01, 1.06)	0.002
Hispanic	1.16 (1.12, 1.2)	<0.001	1.06 (1.02, 1.09)	<0.001
Asian or Pacific Islander/Native American/Other	1.2 (1.14, 1.25)	<0.001	1.09 (1.05, 1.14)	<0.001
Primary payment source				
Medicare	Reference		Reference	
Medicaid	1.18 (1.14, 1.22)	<0.001	1.06 (1.02, 1.09)	<0.001
Private insurance	1.01 (0.99, 1.04)	0.3	0.98 (0.96, 1)	0.031
Self-pay/No charge/Other	0.89 (0.86, 0.93)	<0.001	0.88 (0.84, 0.91)	<0.001
Hospital characteristics				
Hospital region				
Northeast	Reference		Reference	
Midwest	0.84 (0.81, 0.86)	<0.001	0.81 (0.79, 0.83)	<0.001
South	0.96 (0.94, 0.99)	0.003	0.94 (0.92, 0.97)	<0.001
West	0.9 (0.87, 0.93)	<0.001	0.84 (0.82, 0.87)	<0.001
Hospital location and teaching status				
Rural	Reference		Reference	
Urban nonteaching	1.18 (1.14, 1.22)	<0.001	1.15 (1.12, 1.18)	<0.001
Urban teaching	1.35 (1.31, 1.39)	<0.001	1.25 (1.21, 1.28)	<0.001
Hospital bedsize				
Small	Reference		Reference	
Medium	1.06 (1.03, 1.09)	<0.001	1.05 (1.02, 1.07)	<0.001
Large	1.16 (1.13, 1.19)	<0.001	1.15 (1.12, 1.18)	<0.001
Comorbidities				
Anemia	1.3 (1.28, 1.32)	<0.001	1.18 (1.16, 1.2)	<0.001
Autoimmune conditions	1.06 (1.02, 1.09)	<0.001	1.03 (1, 1.06)	0.045
Cancer	1 (0.97, 1.03)	0.84	0.98 (0.96, 1.01)	0.15
Cerebrovascular disease	1.39 (1.33, 1.45)	<0.001	1.14 (1.08, 1.21)	<0.001
Heart failure	1.25 (1.24, 1.27)	<0.001	1.16 (1.15, 1.18)	<0.001
Coagulopathy	1.57 (1.53, 1.61)	<0.001	1.37 (1.34, 1.4)	<0.001
Dementia	1.05 (1.02, 1.07)	<0.001	1 (0.98, 1.03)	0.75
Depression	1.02 (1, 1.04)	0.11	1.02 (1.01, 1.04)	0.009
Diabetes	1.1 (1.08, 1.11)	<0.001	1.02 (1.01, 1.03)	0.005
Hypertension	1.04 (1.02, 1.06)	<0.001	1 (0.98, 1.01)	0.64
Liver disease	1.21 (1.17, 1.26)	<0.001	1.03 (1, 1.07)	0.047

Chronic pulmonary disease	0.86 (0.85, 0.88)	<0.001	0.96 (0.95, 0.98)	<0.001
Obesity	1.18 (1.16, 1.2)	<0.001	1.13 (1.11, 1.15)	<0.001
Paralysis	1.51 (1.45, 1.58)	<0.001	1.27 (1.2, 1.35)	<0.001
Peripheral vascular disease	1.03 (1.01, 1.06)	0.007	1.01 (0.99, 1.03)	0.49
Psychoses	1.12 (1.08, 1.17)	<0.001	1.08 (1.04, 1.12)	<0.001
Pulmonary circulation disease	1.24 (1.22, 1.27)	<0.001	1.16 (1.14, 1.18)	<0.001
Renal disease	1.17 (1.16, 1.19)	<0.001	1.03 (1.01, 1.05)	<0.001
Thyroid disorder	1 (0.98, 1.02)	0.97	1.01 (1, 1.03)	0.084
Valvular disease	1.1 (1.08, 1.12)	<0.001	1.01 (0.99, 1.03)	0.22
Weight loss	1.61 (1.56, 1.65)	<0.001	1.52 (1.49, 1.56)	<0.001

Supplemental Digital Content (Table S6): Predictors of Higher Hospitalization Cost for Acute Respiratory Failure Patients with Delirium.

**Variable**	**Univariable analysis**	***p*-value**	**Multivariable analysis**	***p*-value**
	**IRR (95% CI)**		**aIRR (95% CI)**	
vs. No delirium	1.67 (1.63, 1.72)	<0.001	1.49 (1.46, 1.52)	<0.001
Patient demographics				
Age	0.991 (0.99, 0.991)	<0.001	0.991 (0.99, 0.991)	<0.001
Female vs male	0.9 (0.88, 0.91)	<0.001	0.94 (0.93, 0.96)	<0.001
Race				
White	Reference		Reference	
Black	1.22 (1.18, 1.26)	<0.001	1.08 (1.05, 1.11)	<0.001
Hispanic	1.37 (1.31, 1.43)	<0.001	1.14 (1.11, 1.18)	<0.001
Asian or Pacific Islander/Native American/Other	1.51 (1.43, 1.6)	<0.001	1.23 (1.18, 1.28)	<0.001
Primary payment source				
Medicare	Reference		Reference	
Medicaid	1.29 (1.24, 1.33)	<0.001	0.98 (0.95, 1.01)	0.25
Private insurance	1.15 (1.11, 1.19)	<0.001	0.98 (0.96, 1.01)	0.15
Self-pay/No charge/Other	0.92 (0.87, 0.97)	0.001	0.83 (0.79, 0.87)	<0.001
Hospital characteristics				
Hospital region				
Northeast	Reference		Reference	
Midwest	0.78 (0.75, 0.82)	<0.001	0.77 (0.74, 0.8)	<0.001
South	0.82 (0.79, 0.85)	<0.001	0.8 (0.77, 0.82)	<0.001
West	1.16 (1.1, 1.22)	<0.001	1.08 (1.03, 1.12)	<0.001
Hospital location and teaching status				
Rural	Reference		Reference	
Urban nonteaching	1.21 (1.17, 1.26)	<0.001	1.07 (1.03, 1.11)	<0.001
Urban teaching	1.45 (1.4, 1.51)	<0.001	1.17 (1.13, 1.21)	<0.001
Hospital bed size				
Small	Reference		Reference	
Medium	1.03 (0.99, 1.07)	0.13	1 (0.97, 1.03)	0.87
Large	1.15 (1.11, 1.2)	<0.001	1.07 (1.04, 1.11)	<0.001
Comorbidities				
Anemia	1.31 (1.29, 1.34)	<0.001	1.18 (1.16, 1.2)	<0.001
Autoimmune conditions	1.1 (1.05, 1.16)	<0.001	1.03 (1, 1.06)	0.046
Cancer	1.05 (1.01, 1.08)	0.005	1.02 (0.99, 1.05)	0.17
Cerebrovascular disease	1.5 (1.44, 1.57)	<0.001	1.21 (1.15, 1.26)	<0.001
Heart failure	1.28 (1.26, 1.3)	<0.001	1.23 (1.21, 1.25)	<0.001
Coagulopathy	1.91 (1.85, 1.97)	<0.001	1.57 (1.53, 1.61)	<0.001
Dementia	0.93 (0.91, 0.95)	<0.001	0.95 (0.93, 0.97)	<0.001
Depression	0.95 (0.93, 0.97)	<0.001	0.98 (0.96, 1)	0.014
Diabetes	1.1 (1.08, 1.12)	<0.001	1.03 (1.01, 1.04)	<0.001
Hypertension	0.97 (0.95, 1)	0.029	0.99 (0.97, 1)	0.13
Liver disease	1.34 (1.28, 1.39)	<0.001	1.06 (1.02, 1.1)	0.001

Chronic pulmonary disease	0.73 (0.72, 0.75)	<0.001	0.86 (0.84, 0.87)	<0.001
Obesity	1.17 (1.15, 1.2)	<0.001	1.12 (1.1, 1.14)	<0.001
Paralysis	1.6 (1.53, 1.68)	<0.001	1.22 (1.16, 1.29)	<0.001
Peripheral vascular disease	1.08 (1.04, 1.11)	<0.001	1.05 (1.02, 1.07)	<0.001
Psychoses	1.1 (1.05, 1.15)	<0.001	1.05 (1.01, 1.08)	0.009
Pulmonary circulation disease	1.24 (1.21, 1.27)	<0.001	1.14 (1.12, 1.17)	<0.001
Renal disease	1.2 (1.18, 1.23)	<0.001	1.04 (1.02, 1.06)	<0.001
Thyroid disorder	0.96 (0.94, 0.98)	<0.001	1.01 (0.99, 1.03)	0.28
Valvular disease	1.12 (1.09, 1.15)	<0.001	1.04 (1.02, 1.06)	<0.001
Weight loss	1.73 (1.66, 1.8)	<0.001	1.55 (1.5, 1.59)	<0.001
